# Level of professional ethics awareness and medical ethics competency of dental hygienists and dental hygiene students: the need to add ethics items to the Korean Dental Hygienist Licensing Examination

**DOI:** 10.3352/jeehp.2020.17.34

**Published:** 2020-11-17

**Authors:** Yoon-Sook Hwang, Jong-Hwa Jang, Kyung-Hee Kang, Minji Kim, Jeong-Ran Park, Sohyun Son, Sun-Mi Lee, Da-Yee Jeung, Jung-Eun Ha, Su-Min Hong, Young-Eun Jang

**Affiliations:** 1 Department of Dental Hygiene, Hanyang Women’s University, Seoul, Korea; 2 Department of Dental Hygiene, College of Health Science, Dankook University, Cheonan, Korea; 3 Department of Dental Hygiene, Konyang University, Daejeon, Korea; 4 Dongjak-gu Community Health Center, Seoul, Korea; 5 Department of Dental Hygiene, Baekseok University, Cheonan, Korea; 6 Department of Dental Hygiene, Dongnam Health University, Suwon, Korea; Hallym University, Korea

**Keywords:** Dental hygienists, Licensure, Medical ethics, Professional ethics, Republic of Korea

## Abstract

**Purpose:**

This study aimed to evaluate the level of professional ethics awareness and medical ethics competency in order to assess the potential need for ethics items to be included on the Korean Dental Hygienist Licensing Examination.

**Methods:**

In total, 358 clinical dental hygienists and dental hygiene students completed a structured questionnaire to evaluate their level of ethical awareness and medical ethics competency. The sub-factors of medical ethics were classified into relationships with patients, medical and social relations, and individual specialized fields.

**Results:**

Only 32.1% of participants indicated that they had taken a course on professional ethics in the university curriculum, but 95.2% of respondents considered professional ethics to be important. The overall score for medical ethics competency was average (3.37 out of 5). The score for relationships with patients was 3.75 points, followed by medical and social relations (3.19 points) and individual specialized fields (3.16 points). The level of professional ethics awareness was higher among participants who had taken a course on professional ethics than among those who had not done so or who did not remember whether they had done so.

**Conclusion:**

Dental hygienists were aware of the importance of professional ethics, but their medical ethics competency was moderate. Therefore, medical ethics should be treated as a required subject in the university curriculum, and medical ethics competency evaluations should be strengthened by adding ethics items to the Korean Dental Hygienist Licensing Examination.

## Introduction

### Background/rationale:

The professional ethics of dental hygienists refers to the practice of the doctrines or obligations that should be observed as an oral health professional, and is an act of voluntary will rather than a set of practices encoded by laws or rules. Ethical practices are only possible when the professional ethical standards of dental hygienists are treated as an essential competency and implemented at a high level. The Korean Dental Hygienist Association has enacted a code of professional ethics for dental hygienists and recommends that dental hygienists adhere to this code [1]. However, it is not enough that the code of ethics is merely implemented as a minimum requirement to maintain one’s professional status. A professional ethics course in undergraduate dental hygiene programs in South Korea was offered at only 15 of 74 educational institutions that trained dental hygienists in 2016, and an average of 1.8 hours of education on this topic was conducted [2]. The content of education on ethics also tends to focus on general ethics and work-related ethics, rather than professional ethics for dental hygienists’ jobs; thus, ethics courses related to dental hygienists’ jobs have not yet been established as mandatory courses. Dental hygienists are required to have relatively high levels of professionalism and moral standards because they are granted the status of professionals by society based on a licensing system. Therefore, in order for professional ethics to become an essential competency of dental hygienists, it is necessary to evaluate the ethical competencies essential for dental hygienists through educational programs and national tests.

### Objectives

The purpose of this study was to examine the level of professional ethics awareness and medical ethics competency of clinical dental hygienists and prospective dental hygienists. The specific goals were as follows: first, to determine the general response of dental hygienists to professional ethics; second, to compare medical ethics competency between clinical registered dental hygienists and dental hygiene students; third, to compare the medical ethics competency between participants who had taken a professional ethics course and those who had not done so; and fourth, to compare awareness of professional ethics between registered clinical dental hygienists and dental hygiene students according to whether they had taken a professional ethics course. The results will provide evidence supporting the addition of ethics items to the Korean Dental Hygienist Licensing Examination.

## Methods

### Ethics statement

This study was approved by the Institutional Bioethics Committee of Hanyang Women’s University (IRB approval no., AN01-202005-HR-006-02). Informed consent was obtained from the voluntary participants in the study.

### Study design

This was a survey-based cross-sectional observational study.

### Setting

From May 17, 2020 to May 27, 2020, a structured questionnaire was dispatched to subjects to complete independently in the form of a Google online survey.

### Participants

The participants of this study were clinical dental hygienists with a dental hygienist license and dental hygiene students in Korea. The category of clinical dental hygienists included dental hygienists currently working at an oral health care institution such as a dental clinic or public health center. Recruitment of the clinical dental hygienists for the survey was done by posting about the purpose of the study on a website related to dental hygienists. Dental hygiene students were recruited by arbitrarily selecting 10 universities with dental hygiene departments across the country. Online surveys were distributed to an unspecified number of students at each university. The final number of responses was 177 from clinical dental hygienists and 181 from dental hygiene students.

### Data sources/measurement

The questionnaire was composed of items on the level of awareness of professional ethics and medical ethics competency based on existing research (Supplement 1). Approval and permission were obtained for the use of the tools used in the study from the original researchers [3-5]. Professional ethics awareness consisted of experiences of ethics education, awareness of professional ethics, perceptions of the importance of professional ethics, willingness to participate in professional ethics education, and the desired professional ethics curriculum. Professional ethics perception, importance, and intention to participate in education were measured on a 5-point Likert scale, from “strongly disagree” (1 point) to “strongly agree” (5 points). For medical ethics competency, a questionnaire was administered that was finalized through a Delphi analysis among 36 dental hygienists using a tool developed by Kim et al. [5]. The 53 items analyzed in this study were measured on a 5-point Likert scale, ranging from “strongly disagree” (1 point) to “strongly agree” (5 points), and classified into 3 factors. Factor 1 was termed “relationships with patients” and dealt with patients’ welfare, rights, safety, communication and seeking consent, privacy and confidentiality, telling the truth, and coping with problems in the clinic. Factor 2, named “medical and social relations,” consisted of understanding the theory of medical ethics, professionalism, relationships with colleagues, management of conflicts of interest, medical accidents and disputes, and distribution of medical resources. Factor 3, “individual specialized fields,” included information on public health-related ethics, human research-related ethics, and research integrity. The value of Cronbach’s α was 0.935, indicating the consistency of this research tool.

### Study size

To estimate the adequate sample size for this 2-group comparison study with equal group numbers, G*Power ver. 3.1.9.2 (Heinrich-Heine-Universität Düsseldorf, Düsseldorf, Germany; http://www.gpower.hhu.de/) was used. The minimum estimated sample size was 176 with an actual power of 0.95. The input was 1-tailed analysis, with an effect size of 0.5, an α error probability of 0.05, and an allocation size (N2/N1) of 1. The total sample of 358 was sufficient for this comparative study.

### Statistical analysis

Using IBM SPSS ver. 23.0 for Windows (IBM Corp., Armonk, NY, USA), percentages and descriptive statistics were calculated for characteristics related to professional ethics among clinical dental hygienists and prospective dental hygienists. Differences in medical ethics competency and professional ethics awareness according to the category of subjects and their experience of having taken a professional ethics course were analyzed by Duncan multiple comparisons after the independent t-test and 1-way analysis of variance with the post-test. The significance level of this study was set to α=0.05.

## Results

Raw data of responses from 177 clinical dental hygienists and 181 dental hygiene students are available from Dataset 1 and 2 respectively.

### General response to professional ethics as dental hygienists

The most common perception of “professional ethics” was “high” with 59.8%, followed by “moderate” (12.6%), “very high” (12.6%), and “low” (5.3%). When asked about the importance of professional ethics, 95.2% of the respondents answered that it was important or very important. The largest proportion (40.5%) of the respondents indicated that they did not remember whether they had completed a professional ethics course, followed by those who indicated that they had taken such a course (32.1%) and those who had not done so (27.4%). When asked about the curriculum in which professional ethics education should be conducted, most participants (58.7%) indicated that it should be part of the major, followed by the liberal education curriculum (20.1%) and continuing education (17.3%). Furthermore, 70.2% indicated that they were willing to participate in professional ethics education (Table 1).

### Comparison of medical ethics competency between clinical registered dental hygienists and dental hygiene students

The overall mean score for medical ethics was average (3.37 out of 5). Of the factors of medical ethics, the relationships with patients was the highest (3.75 points,) followed by medical and social relations (3.19 points), and individual specialized fields (3.16 points). In terms of the specific sub-factors, relationships with colleagues had the highest score (4.02 points), followed by patients’ privacy and confidentiality (3.96 points), and telling the truth and dealing with problems in the clinic (3.84 points for both). The sub-factors with below-average scores were understanding and application of the theory of medical ethics (2.43 points), professionalism (2.72 points), and research integrity (2.84 points) (Table 2)

### Comparison of medical ethics competency according to experiences of having taken a professional ethics course

The medical ethics competency (3.55 points) of subjects who had taken a professional ethics course was higher than that of subjects who had not done so (3.30 points) or did not remember (3.27 points) (P=0.001). Those who had taken a professional ethics class had significantly higher scores for all 3 major factors than their counterparts: relationships with patients (3.88 points, P=0.018), medical and social relations (3.40 points, P<0.001), and individual specialized fields (3.36 points, P=0.003) (Table 3).

### Comparison of professional ethics awareness between registered clinical dental hygienists and dental hygiene students according to experiences of having taken a professional ethics course

Among clinical dental hygienists, the score for perceptions of professional ethics was higher for those who had completed a professional ethics course (4.02 points) than those who had not done so (3.56 points) or did not remember (3.63 points) (P<0.001). All respondents answered that they considered professional ethics to be important, with no significant difference according to whether they had taken a professional ethics course. For dental hygiene students, those who had taken a professional ethics course had higher scores for perceptions of professional ethics (4.19 points) than those who had not done so (3.74 points) or were not sure (3.76 points) (P<0.001). The willingness to participate in professional ethics education was higher among both those who had or had not taken a course than among respondents who did not remember whether they had done so (P=0.001). Just as was the case for clinical dental hygienists, all respondents indicated that they considered professional ethics to be important, regardless of whether they had taken a course on the topic (Table 4).

## Discussion

### Interpretation

The results of previous cross-sectional studies on the level of awareness of professional ethics among dental hygienists confirmed that dental hygienists urgently require training in ethical decision-making skills and development of their related competencies through professional ethics education [6,7]. In the present study, 72.4% of respondents said they were aware of the concept of professional ethics. This is slightly higher than a previous report of 66.1% of attendees at a dental hygienist refresher training in 2017 [4], but it is far less than the rate of 93.3% reported among dental hygienists at public health centers [3]. In this study, 95.2% of respondents indicated that they considered professional ethics to be important, but only 32.1% had completed relevant coursework. However, 70.2% of respondents stated that they would agree to participate in ethics education. More than half (58.7%) of the respondents stated the opinion that professional ethics should be included in the major curriculum, and the remaining respondents indicated that it should be incorporated in the liberal arts curriculum or continuing education.

### Comparison with previous studies

You et al. [8] developed an educational program that inculcated values for the social and health care system in order to augment dental hygienists’ values related to the social and health system. Lee and Cheon [4] argued that in order to foster appropriate professional ethics among dental hygienists and to ensure that ethical practices are put into practice, it is necessary to develop and offer courses in professional ethics in dental hygiene education programs and to strengthen professional ethics awareness.

The medical ethics competency of the participants of this study was at an average level (3.37 out of 5 points), confirming the need to strengthen ethics education. Lee and Han [9] argued that since dental hygienists are a human-centered profession, it is important to establish values that place a higher importance on professional ethics and bioethics than many other professions. Several developed countries have codes of ethics for dental hygienists [10,11], as exemplified by the Korean Dental Hygienist Association’s code of ethics [1]. In the World Dental Hygienist Federation’s code of ethics, the core values that dental hygienists must have are integrity and respect [12]. These core values can be realized through ethical behaviors that a dental hygienist should show, and the standards of conduct are categorized into 4 categories (dental hygienists and people/society; work performance; co-workers; professional behavior). Thus, this code not only presents standards of ethical behavior between the dental hygienist and the patient, but also refers to the standards of behavior of dental hygienists as members within the broader community. In addition to complying with regulations, it is also important to collaborate with colleagues in related fields. In addition, the field of dental hygiene should be improved by continuing to cultivate the latest knowledge and technology related to dental hygiene. The medical ethics competencies investigated in this study were classified into relationships with patients, medical and social relations, and individual specialized fields, but guidelines for evaluating ethical competencies related to items that address unique aspects of the work of dental hygienists are needed.

In 2016, the Korea Health Personnel Licensing Examination Institute published “Research on policy proposals for fostering and producing excellent human resources” for 15 occupations, and for most occupations, including dental hygienists, argued that it was necessary to strengthen ethics education and assessments [2]. In “A study on the application of national examination medical ethics for health care workers” [13], representatives from the associations of dental hygienists, emergency medical technicians, and occupational therapists stated that medical ethics education was very necessary. The Ministry of Health and Welfare revised the 2017 Health and Medical Personnel License Report and Refresher Education Guideline and newly established medical ethics as a mandatory subject for continuing education. This revision also addressed compliance with medical-related laws [14].

Currently, the occupations for which medical ethics questions are asked in the national examination for medical personnel and medical technicians in Korea are medical doctors, dentists, oriental medicine doctors, pharmacists, nurses, and occupational therapists [13].

### Limitations

To reflect the results of this study in the Korean Dental Hygienist Licensing Examination, it will be necessary to prepare a guideline that can measure ethical competencies related to unique aspects of dental hygienists’ work responsibilities.

### Conclusion

This study evaluated the medical ethics competencies of dental hygienists based on their experience of having taken a professional ethics course at a time when the importance of ethical values of health care professionals is highlighted, and the findings provide basic data that can be reflected in the national examination for dental hygienists. In addition, the findings of this study can contribute to enhancing the directions of ethical education for dental hygienists. Further research is required to develop detailed items and guidelines for medical ethics competency that can be specifically reflected in the national examinations. In addition, it is suggested that universities should develop and offer ethics courses in the dental hygiene major curriculum, and that for clinical dental hygienists, policy steps should be taken to further strengthen ethics education through continuing education.

## Figures and Tables

**Table 1.** Characteristics related to the professional experience of dental hygienists

Characteristic	Category	No. (%)
Type	RDH	177 (49.4)
	DHS	181 (50.6)
Recognition of professional ethics	Very high	45 (12.6)
	High	214 (59.8)
	Moderate	80 (22.3)
	Low	19 (5.3)
Importance of professional ethics	Strongly agree	154 (43.0)
	Agree	187 (52.2)
	Neutral	16 (4.5)
	Disagree	1 (0.3))
Experience of having taken professional ethics courses	Yes	115 (32.1)
	No	98 (27.4)
	Do not remember	145 (40.5)
Recognition of the field of	Major curriculum	210 (58.7)
professional ethics curriculum		
	Liberal education	72 (20.1)
	Continuing education	62 (17.3)
	Other	14 (3.9)
Intention to participate in professional ethics courses	Strongly agree	60 (16.8)
	Agree	191 (53.4)
	Neutral	86 (24.0)
	Disagree	15 (4.2)
	Strongly agree	6 (1.7)
	Other	17 (4.7)

RDH, registered clinical dental hygienist, DHS, dental hygiene student.

**Table 2.** Comparison of medical ethics competencies between clinical registered dental hygienists and dental hygiene students

Characteristic	Medical ethics competencies	Cronbach’s α
Relationships with patients	3.75±0.59	0.949
Patient welfare, rights and safety	3.35±0.69	0.924
Communication and seeking consent	3.78±0.63	0.924
Patient privacy and confidentiality	3.96±0.74	0.928
Telling the truth	3.84±0.74	0.926
Coping with problems in the office	3.84±0.74	0.927
Medical and social relations	3.19±0.70	0.909
Understanding and application of medical ethics theory	2.43±1.07	0.932
Professionalism	2.72±0.95	0.924
Relationship with colleagues	4.02±0.72	0.928
Conflicts of iInterest	3.15±0.83	0.921
Medical accidents and disputes	3.32±0.86	0.923
Distribution of medical resources	3.51±1.00	0.925
Individual specialized fields	3.16±0.81	0.928
Ethics related to public health	3.39±0.83	0.922
Ethics related to human research	3.24±0.84	0.922
Research integrity	2.84±1.13	0.929
All	3.37±0.63	0.935

Values are presented as mean±standard deviation, unless otherwise stated. Min value=1.00, max value=5.00.

**Table 3.** Assessment of medical ethics competencies based on experience of having taken a professional ethics course

Characteristic	Experience of having taken a professional ethics course			P-value
	Yes (n=115)	No (n=98)	Do not remember (n=145)	
Relationships with patients	3.88±0.61^a^	3.70±0.63^b^	3.69±0.55^b^	0.018
Patient welfare, rights and safety	3.62±0.68^a^	3.24±0.71^b^	3.22±0.62^b^	<0.001
Communication and seeking consent	3.91±0.63^a^	3.66±0.68^b^	3.74±0.58^b^	0.013
Patient privacy and confidentiality	4.06±0.72	3.94±0.80	3.88±0.70	0.167
Telling the truth	3.89±0.73	3.77±0.74	3.84±0.78	0.460
Coping with problems in the office	3.93±0.75	3.87±0.80	3.74±0.67	0.127
Medical and social relations	3.40±0.71^a^	3.11±0.72^b^	3.08±0.64^b^	<0.001
Understanding and application of medical ethics theory	2.73±1.16^a^	2.30±1.06^b^	2.28±0.95^b^	0.001
Professionalism	3.06±0.96^a^	2.52±1.02^b^	2.57±0.80^b^	<0.001
Relationship with colleagues	4.11±0.69	3.93±0.76	4.00±0.70	0.206
Conflicts of interest	3.33±0.82^a^	3.13±0.85^ab^	3.00±0.79^b^	0.006
Medical accidents and disputes	3.49±0.86^a^	3.35±0.89^ab^	3.17±8.20^b^	0.011
Distribution of medical resources	3.68±0.88^a^	3.39±1.05^ab^	3.46±1.04^b^	0.076
Individual specialized fields	3.36±0.79^a^	3.09±0.85^b^	3.04±0.78^b^	0.003
Ethics related to public health	3.64±0.74^a^	3.30±0.90^b^	3.25±0.80^b^	<0.001
Ethics related to human research	3.40±0.84^a^	3.18±0.89^b^	3.14±0.80^b^	0.040
Research integrity	3.05±1.15^a^	2.78±1.11^ab^	2.72±1.11^b^	0.047
All	3.55±0.64^a^	3.30±0.66^b^	3.27±0.58^b^	0.001

Values are presented as mean±standard deviation, unless otherwise stated. a,b show by different characters letters that there were significant differences by 1-way ANOVA analysis of variance and the Duncan analysis test at alpha=0.05

**Table 4.** Comparison of professional ethics awareness between RDH and DHS according to their experiences of having taken a professional ethics course

Characteristic	Experience of having taken a professional ethics course			P-value
	Yes (n=115)	No (n=98)	Have no memory (n=145)	
RHD (n=177)				
Recognition of professional ethics	4.02±0.58^a^	3.56±0.78^b^	3.53±0.70^b^	<0.001
Importance of professional ethics	4.46±0.58	4.25±0.67	4.23±0.56	0.085
Intention to participate in professional ethics courses	3.88±0.94	3.79±0.95	3.71±0.82	0.590
DHS (n=181)				
Recognition of professional ethics	4.19±0.50^a^	3.74±0.82^b^	3.76±0.73^b^	<0.001
Importance of professional ethics	4.59±0.50	4.40±0.55	4.37±0.58	0.055
Intention to participate in professional ethics courses	4.02±0.66^a^	3.94±0.80^a^	3.57±0.74^b^	0.001

a,b show by different characters letters that there were significant differences by 1-way ANOVA analysis of variance and the Duncan analysis test at alpha=0.05. RDH, registered clinical dental hygienist; DHS, dental hygiene student.

## Footnotes

**Data availability** Data files are available from: https://doi.org/10.7910/DVN/GU4QKY. Dataset 1. Raw data of responses from 177 clinical dental hygienists. Dataset 2. Raw data of responses from 181 dental hygiene students.

## Supplementary materials

Supplement 1. Survey questionnaire

Supplement 2. Audio recording of the abstract.
