# Supplementary material for: Level of professional ethics awareness and medical ethics competency of dental hygienists and dental hygiene students: the need to add ethics items to the Korean Dental Hygienist Licensing Examination
Source: J Educ Eval Health Prof. 2020 Nov 17;17:34. doi: 10.3352/jeehp.2020.17.34 (PMC7758182; doi:10.3352/jeehp.2020.17.34)
Supplement: Supplementary file 3 — Supplement 1. Survey questionnaire [file jeehp-17-34-suppl1.docx]

**Dental Hygiene Student**

**I. Respondent characteristics**

1. When was your birth year? (Year)

2. Gender: ① Male ② Female

3. What grade are you currently in?

① 1st grade ② 2nd grade ③ 3rd grade ④ 4th grade

4. What is the school system at your university?

① 3 years ② 4 years ③ Intensive major ④ Other

5. Where is your university located?

① Seoul, Gyeonggi, Incheon ② Chungcheong area ③ Jeolla area

④ Gyeongsang area ⑤ Gangwon area ⑥ Jeju

7. How much do you know about the professional ethics that dental hygienists should follow?

① I know very well ② I know ③ I fairly know ④ I know little ⑤ I don't know

8. Do you think vocational ethics training is important to dental hygienists?

① Very much ② Yes ③ Fairly ④ No ⑤ Not at all

9. Do you think dental hygienists need professional ethics training?

① Very much ② Yes ③ Fairly ④ No ⑤ Not at all

10. Do you have any experience in vocational ethics training for dental hygienists?

① Yes ② No ③ Not sure

11. Where do you think dental hygienist vocational ethics training should take place?

① University major curriculum

② University liberal arts curriculum

③ University comparative course curriculum

④ Association or society refresher training

⑤ Other

12. Would you like to take a course on vocational ethics education in your university curriculum?

① Very much ② Yes ③ Fairly ④ No ⑤ Not at all

**Registered Dental Hygienist**

1. When was your birth year? (Year)

2. Gender: ① Male ② Female

3. Working experience: Years and months

4. Married or not?

① Married ② Single ③ Other

5. What is your final education level?

① Junior college graduate ② University graduate ③ Graduate school graduate ④ Other

6. What type of work are you working for?

① Dental clinic ② Dental hospital ③ University (general) hospital ④ Public health center

⑤ Other

7. Where are you working?

① Seoul, Gyeonggi, Incheon ② Chungcheong area ③ Jeolla area ④ Gyeongsang area

⑤ Gangwon area ⑥ Jeju

8. How much do you know about the professional ethics of a dental hygienist?

① I know very well ② I know ③ I fairly know ④ I know little ⑤ I don't know

9. Do you think vocational ethics training is important for dental hygienists?

① Very much ② Yes ③ Fairly ④ No ⑤ Not at all

10. Do you think dental hygienists need vocational ethics training?

① Very much ② Yes ③ Fairly ④ No ⑤ Not at all

11. Do you have any experience in vocational ethics training for dental hygienists?

① Yes ② No ③ Not sure

12. Where do you think dental hygienist vocational ethics training should take place?

① University major curriculum

② University liberal arts curriculum

③ University comparative course curriculum

④ Association or society refresher training

⑤ Other

13. Would you like to take a course on vocational ethics education as a refresher education at an association or conference?

① Very much ② Yes ③ Fairly ④ No ⑤ Not at all

**II. Relationship with the patient**

❏ This is about the relationship with patients in professional ethics. Please respond with ‘√’ to the degree of consent for each item.

| **Item** | | Strongly disagree ~ Strongly agree | | | | |
| --- | --- | --- | --- | --- | --- | --- |
| A | 1. The best interests of patients can be identified based on medical interests and patient preferences. | 1 | 2 | 3 | 4 | 5 |
|  | 2. Can list the overall obligations of the dental hygienist in the best interests for the patient |  |  |  |  |  |
|  | 3. Can understand and manage human, social and institutional elements that can guarantee the best interests for patients |  |  |  |  |  |
|  | 4. Can explain the overall contents of the patient, such as the right to receive the best treatment, the right not to be discriminated against, and the patient's right to self-determination. |  |  |  |  |  |
|  | 5. Can explain the basis of the patient's rights, such as the right to receive the best treatment, the right not to be discriminated against, and the patient's right to self-determination. |  |  |  |  |  |
|  | 6. Can explain the principles and priorities of determining patient rights to be applied in individual circumstances |  |  |  |  |  |
|  | 7. Can explain the overall obligation of the dental hygienist to patient safety |  |  |  |  |  |
|  | 8. Ability to understand and manage human and institutional factors that can cause patient safety incidents |  |  |  |  |  |
|  | 9. Can explain the obligations related to the disclosure of patient safety events and the reporting system and procedures |  |  |  |  |  |
| B | 1. Can listen to the opinions of the patient, respect the position, and recognize and empathize with the patient's thoughts, feelings, and value system |  |  |  |  |  |
|  | 2. Collect necessary information from patients, manage them safely, and share information to agree on future plans. |  |  |  |  |  |
|  | 3. If the patient's doctor is different from the caregiver or medical staff, the reason for prioritizing the patient's opinion can be explained. |  |  |  |  |  |
|  | 4. The patient's consent can be obtained by providing all information related to the patient and sufficiently explaining it. |  |  |  |  |  |
|  | 5. If the patient's decision-making ability is not intact (children, minors, dementia patients, mentally ill patients, etc.), the criteria for determining the qualifications of a representative and consent can be applied. |  |  |  |  |  |
|  | 6. Identify economic/social factors that may affect patient spontaneity, minimize them, and obtain consent |  |  |  |  |  |
|  | 7. If you intend to use medical information related to patients for purposes other than diagnosis and treatment, consent based on sufficient information can be obtained. |  |  |  |  |  |
|  | 8. In situations in which the confidentiality of the patient is an exception, sufficient information can be provided to the patient and consent can be obtained from the patient. |  |  |  |  |  |
|  | 9. Dentist's explanations and patient's decision-making (consent or rejection) can be recorded. |  |  |  |  |  |
| C | 1. Can keep confidential information related to certain diseases and treatment, including personal information obtained from patients during treatment, including minors |  |  |  |  |  |
|  | 2. Understand patient confidentiality exceptions, understand relevant laws and information, and provide sufficient information to patients. |  |  |  |  |  |
| D | 1. Providing bad news to the patient according to the proper procedure, explaining it appropriately, and making a plan for the future by talking on the basis of empathy |  |  |  |  |  |
|  | 2. In the event of a medical error, it can be disclosed and communicated for apology according to the principle. |  |  |  |  |  |
| E | 1. Can respond appropriately to the needs of examination or treatment of patients (family or guardians) who are contrary to medical knowledge |  |  |  |  |  |
|  | 2. Able to recognize and respond appropriately to financial or affectionate relationships, including gifts that threaten patient-doctoral relationships. |  |  |  |  |  |

**III. Medical and Social Relations**

❏ This is about medical and social relations' in occupational ethics. Please respond with ‘√’ to the degree of consent for each item.

| **Item** | | Strongly disagree ~ Strongly agree | | | | |
| --- | --- | --- | --- | --- | --- | --- |
| F | 1. Can list and explain 4 principles of medical ethics | 1 | 2 | 3 | 4 | 5 |
| G | 1. Can list and explain the concepts and components of professionalism |  |  |  |  |  |
|  | 2. Can explain job autonomy and social responsibility |  |  |  |  |  |
|  | 3. Can explain the reasons for the need for a code of ethics for dental hygiene professionals |  |  |  |  |  |
| H | 1. Can express understanding and respect for various roles and responsibilities with fellow dental hygienists |  |  |  |  |  |
|  | 2. Can listen to the opinions of fellow dental hygienists and express courtesy and consideration |  |  |  |  |  |
|  | 3. Maintain a partnership with dentists and can provide professional opinions and cooperate. |  |  |  |  |  |
|  | 4. Understand team health and can explain the roles and responsibilities of dental hygienists in ensuring teams function effectively. |  |  |  |  |  |
|  | 5. Create and maintain a positive work environment free from discrimination and harassment |  |  |  |  |  |
| I | 1. Can explain the concept of a conflict of interest |  |  |  |  |  |
|  | 2. Can adequately cope with conflicts of interest arising from the health care system |  |  |  |  |  |
|  | 3. Able to respond appropriately to conflicts of interest arising in the research process |  |  |  |  |  |
|  | 4. Can define unfair advantage and explain the basis for injustice |  |  |  |  |  |
|  | 5. Types of unfair gain acquisition can be listed |  |  |  |  |  |
|  | 6. Understand and explain ethical issues when using mass media |  |  |  |  |  |
| J | 1. Can explain the causes of increased conflict with patients |  |  |  |  |  |
|  | 2. Can understand the concept of medical accidents, disputes and litigation |  |  |  |  |  |
| K | 3. Can explain prohibition of discrimination on the grounds of race and ethnicity, age and sex, occupation and position, economic status, ideology and religion, and social reputation |  |  |  |  |  |

**IV. Individual Specialty Field**

❏ This is about individual specialty field' in vocational ethics. Please respond with ‘√’ to the level of consent for each item.

| **Item** | | Strongly disagree ~ Strongly agree | | | | |
| --- | --- | --- | --- | --- | --- | --- |
| L | 1. Can explain the roles and responsibilities of individual, public, national and dental hygiene professions in public health | 1 | 2 | 3 | 4 | 5 |
|  | 2. Understand the roles and responsibilities of dental hygienists in public health-related work affecting community and international health. |  |  |  |  |  |
|  | 3. Can explain legal and ethical principles of infectious disease management |  |  |  |  |  |
|  | 4. Understand patient confidentiality exceptions and provide sufficient information to patients in those circumstances. |  |  |  |  |  |
|  | 5. Understand individual responsibilities and limitations in health promotion, the role of government and ethical issues regarding their limitations, and explain the role of dental hygienists. |  |  |  |  |  |
| M | 1. Can explain the difference between treatment and research |  |  |  |  |  |
|  | 2. Can explain the purpose and function of the institutional bioethics committee |  |  |  |  |  |
|  | 3. Subjects can be appropriately protected according to the guidelines related to research. |  |  |  |  |  |
|  | 4. All information related to the study is provided and sufficiently explained so that the consent of the study participants can be obtained based on this. |  |  |  |  |  |
|  | 5. Efforts can be made to identify factors that may affect the spontaneity of research participants and minimize them. |  |  |  |  |  |
| N | 1. Be able to explain the concept of research integrity |  |  |  |  |  |

♡ Thank you for responding to the end ♡
